# Stability Efficiencies of POSS and Microalgae Extracts on the Durability of Ethylene-Propylene-Diene Monomer Based Hybrids

**DOI:** 10.3390/polym14010187

**Published:** 2022-01-03

**Authors:** Traian Zaharescu, Carmen Mateescu

**Affiliations:** INCDIE ICPE-CA, 313 Splaiul Unirii, 03138 Bucharest, Romania

**Keywords:** EPDM, POSS, microalgae extracts, hybrids, accelerated degradation, irradiation, radiation processing

## Abstract

The EPDM (ethylene-propylene-diene monomer) hybrids with improved thermal and radiation strengths containing 1 and 5 phr of polyhedral oligomeric silsesquioxane (vinyl-POSS, Ov-POSS) and/or 2 phr of microalgae (*Chlorella vulgaris* (*CV*) and *Spirulina* *platensis* (*SP*)) powders were investigated in respect to their thermal stability after γ-irradiation. The material durability under accelerated degradation was qualified by chemiluminescence and gelation, which prove the contribution of inorganic filler and microalgae extracts on the increase of hybrid thermal stability, as well as the interaction between added components (POSS and *CV* or *SP*). The activation energies and the durabilities under accelerated degradation were calculated, indicating their suitable usage as appropriate materials in various applications. The reported results indicate the improvement effect of both microalgal powders on the oxidation strength, but the contribution of *Spirulina* *platensis* grabs attention on its efficient effects upon the prevention of degradation under accelerated aging conditions. The thermal performances of the tested EPDM based hybrids are remarkably ameliorated, if the certain formulation includes Ov-POSS (5 phr) and *Spirulina* *platensis* (2 phr), certifying its suitability for the pertinent applications.

## 1. Introduction

As it may be noticed from the literature, the investigations on the extension of material life generate several papers presenting interesting faces of material properties. Most of them, suitable for general or specific applications, disregard the characterization of the material response to the action of various degradation conditions due to energetic transfer onto products. In the cases of polymer materials used in food preservation and handling, medical and pharmaceutical wear, engineering products, and automotive, aircraft, or rail materials, the stability studies provide valuable data for manufacturers and customers and create favorable conditions for the application of heat and radiation sterilizations.

The considerable interest on the polyhedral oligomeric silsesquioxane (POSS) hybrids for the extension of the potential application areas of plastics manufacture is inspired by many papers [1,2,3] as the appropriate candidates for high performance materials. The spatial structure of POSS is an opportune basic solution for the amelioration of thermal properties [4,5], as well as at the improvement of the durability, of host material [6]. The outstanding benefit of POSS hybrids is its environmental friendliness because their presence does not generate hazardous by-products [7,8], and it has a certain availability for functionalization [9].

Among the promising and compatible inorganic fillers, POSS shows a particular cage effect in respect to the fragments resulting from the degradation of polymers because the large holes created by the scission of (SiO_1.5_) moieties allow the penetration of free radicals into the special structure of POSS. It was previously demonstrated [10] that these structures are easily penetrated by peroxyl radicals. Consequently, the oxidative degradation of polymer matrix is delayed. The antioxidant feature of POSS have been scarcely reported, though several other physical effects have been regularly highlighted [1,11,12,13]. The previous assays on the contribution of POSSs to the amelioration of physical properties confirm the availability of this class kind of fillers for the interaction between these inorganic particles and macromolecular chains or segments, dropping down the shortcomings of fast thermal aging. A comprehensive analysis on the concern of POSS in the degradation of polymer support, such as polyurethane foam [14] or PBAT/PLA [15], emphasizes the implication of filler in the stability of polymer substrate.

**Figure 1:**
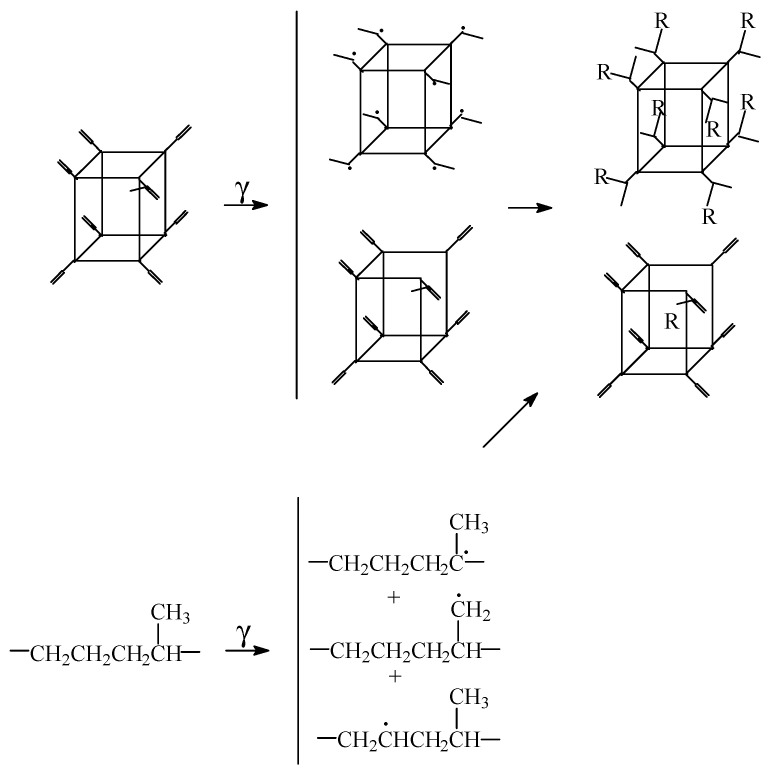
Proposed scheme for the stabilization mechanism of POSS in the γ-irradiated EPDM.

The chemical modifications induced by the high energy irradiation are summarized in the chain fragmentation, when the generation of hydroperoxides by the oxidation of free radicals initiates the self-catalyzed progress of degradation. However, the presence of POSS nanoparticles allows the crosslinking of polymer [16] due to the cage effect in respect to the scavenging degradation precursors and blocks the propagation step of oxidation. In several suggestive cases, the nanoparticles of POSS act as delayer agent in the aging polymers [17,18]. In the cases where vinyl POSS is added in ethylene-propylene-diene monomer (EPDM), the scheme of the interaction between POSS structure and surrounding polymer is suggestively presented in Figure 1. The highest concentration of radicals is provided by the polypropylene segments because the main radicals appeared in damaging EPDM are formed by the removing of proton from the highly substituted carbon atoms [19] (upper structure from Figure 1). The splitting of norbornene contained in EPDM macromolecules may be also considered as a source of reactive positions in irradiated material. The scavenging of free radicals affords a higher stability of polymer by the crosslinking of recombined intermediates [3]. The significant outcomes on the saving of materials and energy are obtained as the result of the gelation process.

The degradation of EPDM has been extensively studied [20,21,22,23] due to its functional performances and abroad spectrum of industrial and commodities applications. The manufacture of high-performance EPDM products must avoid the excessive consumption of oxygen because the generation of peroxyl radicals, their decay by the autocatalytic degradation (the reaction P· + POO·) or bimolecular recombination (POO· + POO·) [24], promotes degradation. As it was previously demonstrated for the oxidation of EPDM, the heating rate may profoundly influence the thermal resistance of this polyolefin [25]. The large range of activation energies from 23 kJ mol^−1^ up to 428 kJ mol^−1^ are obtained using different calculation kinetic procedures based on the various computation models and on the different conversion degrees which were taken into consideration. This situation prompts a new approach on the energetic evaluation of degradation/stabilization behavior of EPDM starting from the oxidation of various formulations of protected EPDM.

The antioxidant activity of algae is commonly demonstrated by their addition in food as the natural component of biological protection. The low irradiation doses less than 1 kGy are usually concerned with the generation of free radicals causing the damage of radiation bio-oxidation [26]. The extended applications cover the therapeutic usage [27]. However, the investigations on the contribution of algal content upon the improvement of the thermal or radiation stabilities in polymers were scarcely achieved in spite of their high content of polyphenols [28]; the antioxidant activity of microalgae was previously proved for the safety preservation of the dietary proteins [29]. In this study, the synergistic contributions of POSS and microalgal extracts (*Chlorella vulgaris* and *Spirulina* *platensis*) on the protection of ethylene-propylene-diene monomer against oxidation are discussed. The expected effects of the present additives are related to the decrease in the oxidation rates, as well as the effective interaction between the coupling powders, whose concern may be characterized by the activation energies and corresponding durabilities. These innovative antioxidant systems consisting of an inorganic compound and natural blends of polyphenols provide an excellent target for the manufacture of ecological nanomaterial products assisted by γ-processing. Their application areas may be broadened over medical wear, food packaging and beverage and drug bottles, toys, gaskets, O-rings and pipes in the food industry, or medical equipment sustained by the prevention of oxidation degradation and a good biocompatibility.

The present results, which illustrate the appropriate prevention of degradation in ethylene-propylene elastomer by polyhedral oligomeric silsesquioxane, complete the previously reported information in the cases of polyurethanes or epoxy resins, as well as extend the studies on the blends consisting of EPDM and hydrogenated acrylonitrile-butadiene rubber [30] or with various additives [31,32]. Nevertheless the association of these studied microalgae powders with an inorganic filler, POSS, is an original version of the degradation inhibition applicable to several materials.

## 2. Materials and Methods

### 2.1. Materials

Raw ethylene-propylene diene monomer was manufactured by LANXESS (The Heerlen—former DSM Elastomers, The Netherland) as KELTAN 8340/A type, whose diene (5-ethylene-2-norbornene) content was initially 5 phr. The used microalgal extracts of *Chlorella vulgaris* and *Spirulina* *platensis* were provided by R & D National Institute of Chemistry and Petrochemistry (Bucharest, Romania). They were added in the present formulations as the received component. Polyhedral oligomeric silsesquioxane (1-vinyl-3,5,7,9,11,13,15-isobutylpentacyclo-[9.5.1.1^3.9^.1^5.15^.1^7.13^]octasiloxane) as vinyl-POSS (Ov-POSS) was purchased from Sigma-Aldrich (Saint Louis, MO, USA). *Chlorella* sp. microalgae in powder form has the following chemical composition (*w/w*%): fats 11%, of which 2% are saturated forms; carbohydrates 25%, of which sugars are 1%; proteins 56%; and natrium chloride 0.1%, while the powder of *Spirulina* sp. microalgae contains fats 8%, of which 1% are saturated structures; carbohydrates 13% of which sugars are 2%; proteins 67%; and natrium chloride 1.1%.

### 2.2. Sample Preparation and Irradiation

The EPDM samples were prepared from the pre-purified elastomer, assuring the elimination of manufacturing additives by the dissolution of neat material in chloroform and the separation of insoluble fraction by filtration through Whatman paper. For the preparation of daughter solutions, the protector powders were individually added. They were obtained by the addition of either vinyl-POSS, *Chlorella vulgaris* and *Spirulina platensis*, or couples of them (inorganic filler/microalgal extract).

All samples contain two concentrations of vinyl-POSS (1 and 5 phr), while the microalgal extracts have a concentration of 2 phr. From these separate solutions, the liquids were poured into round aluminum trays. The dried, thin pellicles were obtained by solvent evaporation at room temperature. For the accelerated degradation, the samples were γ-exposed in irradiation machinery (Ob Servo Sanguis, Hungary) equipped with ^60^Co source in air at room temperature. The applied dose rate was 0.6 kGy h^−1^. The γ-exposure was accomplished by the permanent sample rotation that provides a homogenous irradiation. All the samples were handled in a desiccator for avoiding the action of environmental agents to modify the sample structuring between different stages of investigation.

The γ-exposure of samples was accomplished as a proper manner for an accelerated degradation, where the concentration of scission fragments is high enough for reliable experiments of durability.

### 2.3. Characterization

#### 2.3.1. Chemiluminescence (CL)

**Figure 2:**
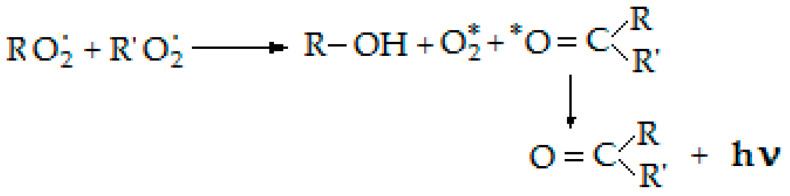
CL mechanism describing the generation of measuring photons during the degradation of polymers.

For the evaluation of oxidation degrees two investigation techniques of chemiluminescence were applied. A chemiluminescence spectrometer (LUMIPOL 3–Slovak Academy of Science, Bratislava) was used to characterize the progress of oxidation by isothermal and non-isothermal measurements [33]. The basic process by which the photon is emitted is presented in the following scheme, in Figure 2.

For the isothermal determinations, several temperatures (150 °C, 160 °C, 170 °C, and 180 °C) were selected, considering the convenient oxidation rates. For the non-isothermal determinations, four heating rates (3.7, 5.0, 10.0, and 15.0 °C min^−1^) were applied. The temperature readings have an average error of ± 0.5 °C. The progress of oxidation can be watched because the CL intensity is proportional with the concentration of peroxyl radicals, the former precursor of oxygenated degradation products [34]. The activation energies involved in the oxidation of nonirradiated samples and the durability of the formulations for all applied doses were calculated from the oxidation induction times (OIT), one of the main kinetic parameters of isothermal degradation using Arrhenius Equation (1).

(1) $$t_{x}=t_{ref}\;exp\left[-\frac{E_{a}}{R}\left(\frac{1}{T_{ref}}-\frac{1}{T_{x}}\right)\right].$$

The evaluation of durability was carried out using the same relationship, where *t_x_* and *t_ref_* are the durability times for any temperature and reference, respectively. *E_a_* is the activation energy required for oxidation, and *R* is the gas constant (8.314 kJ kmol^−1^ K^−1^). *T_ref_* is the reference temperature (170 °C) for the evaluation of durability.

Due to the high reproducibility of CL spectra, only one spectrum was recorded for each composition under foreseen measurement conditions.

For the evaluation of faith degree for the experimental measurements, i.e., the CL intensity versus temperature, five experiments for the inflexion point of the oxidation (an illustrative kinetic parameter for the evaluation of polymer degradation by chemiluminescence assay during the degradation of neat polymer at 150 °C) were done. The found values expressed in °C were: 171, 172, 168, 166, 172. The standard deviation was σ = 3.1.

#### 2.3.2. Crosslinking Evolution

The evaluation of crosslinking levels was conducted by dissolution of soluble fraction contained in polymer phase. This procedure was accomplished by the boiling of copper fabric bags containing polymer hybrids (about 0.2 g each) in toluene in a Soxhlet unit for 24 h. For each point, three bags were processed. The bags were weighted before and after boiling with an analytical scale (error ± 0.1 mg). Each value of insoluble fraction was obtained from three similar samples. The insoluble fraction evaluated with an error of ±0.7%, *p*, was calculated with the Equation (2):

(2) $$p\left(\%\right)=\frac{m_{f}-m_{0}}{m_{i}-m_{0}}\cdot \;100,$$

where *m_i_* and *m_f_* are the initial and final weights of bags containing sample (before and after irradiation, respectively), while *m_o_* is the weight of empty bag. The polymer mass (*m_i_* or *m_f_*) was reconsidered after the abstraction of fillers contributions from the weight listed on the scale screen.

For the characterization of the evolution of crosslinking by the values of *P*(*x*)/*Q*(*x*), the ratio between the scission radiochemical yield, *P*(*x*), and crosslinking radiochemical yield, *Q*(*x*), and the insoluble fractions (*p*) figures were converted into sol fraction, *S*, by their abstraction from unity. From the application of Charlesby-Pinner relationship (Equation (3)) [35], the intercept of linear dependence provides the expected ratio, where *q*_0_, *U*_2,0_, and *D* are initial crosslinking density, initial degree of polymerization, and irradiation dose (kGy), respectively.

(3) $$S+S^{1/2}=\frac{P\left(x\right)}{Q\left(x\right)}+\frac{2}{q_{0}U_{2,0}}\frac{1}{D}.$$

The representation of sol values provided the equations of crosslinking from which the slopes were calculated.

#### 2.3.3. Scanning Electron Microscope

SEM assay on the Ov-POSS powder was accomplished with a scanning electron microscope SEM Aurega (Carl Zeiss, Stuttgart, Germany), type field emission. All presented images have the magnification of 20,000.

## 3. Results

The consequences of radiation processing of ethylene-propylene elastomers are satisfactorily explained by a radical mechanism [19,22,35,36,37,38]. The competition between scission and crosslinking influences the sample morphology and oxidation level. While the mechanical behavior is well defined in relation with the progress of oxidation [20], the protection against oxidation takes multiple faces. The conversion of free radicals into peroxyl moieties can be restricted by blocking former intermediates by antioxidants [39], by cage scavenging with POSS [40] or by inorganic oxide fillers [41]. The growth of gel fraction by random crosslinking is initiated by native hydrocarbon fragments, while the oxidative degradation is propagated by hydroperoxide intermediates which are rearranged to form final oxygen containing degradation products [42]. The kinetic approach on the degradation of ethylene-propylene elastomers stated that the molecular scission followed by the competitive crosslinking versus oxidation explained the structural changes of ethylene-propylene elastomers, where the major role of diene in the development of oxidation state is determinative in detail [36,43]. During γ-irradiation, the oxygen consumption depends linearly on the square root of O_2_ [24]. Accordingly, the contribution of diffused oxygen during degradation can be minimized by the addition of the appropriate compounds (antioxidants and/or fillers) that prevent material aging.

### 3.1. Radiation Degradation of Additives

#### 3.1.1. Ov-POSS

**Figure 3:**
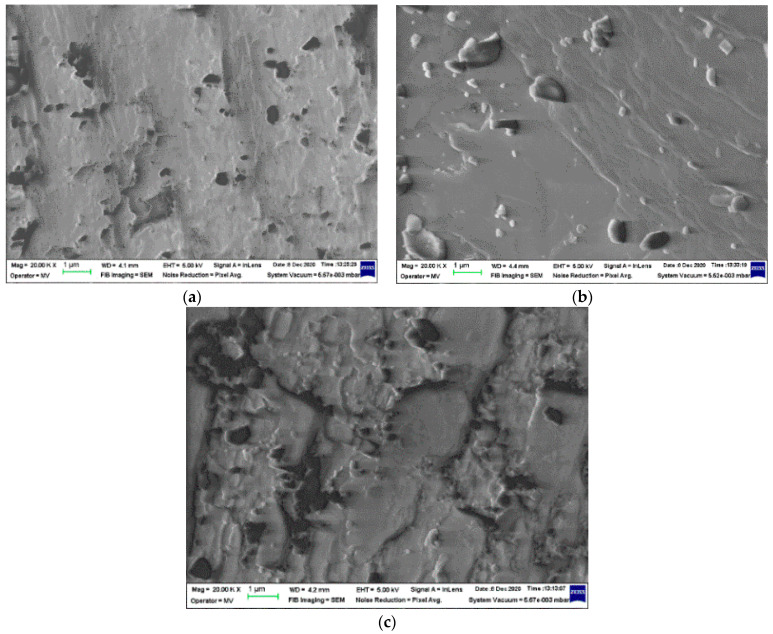
SEM images of Ov-POSS al various radiation processing doses. (**a**) 0 kGy; (**b**) 25 kGy; (**c**) 50 kGy. Magnification 20,000.

The inorganic structure of Ov-POSS seems to be stable over a large range of degradation conditions. However, this feature of the polyhedral oligomeric silsesquioxane was scarcely investigated [10]. Our SEM assay on Ov-POSS powder (Figure 3) reveals the evolution of crystals over a large dose range, when the morphology is changed.

The polymer crosslinking progresses by the contribution of unsaturation existing in vinyl structure of Ov-POSS or in the EPDM macromolecules as the added diene (5-ethylidene 2-nornbornene), where initial concentration in the major component is 5 phr. The hardening of crystals is associated with their damage, by which the break and detachment of crystal small pieces lead to the worsening of stabilization efficiency.

**Figure 4:**
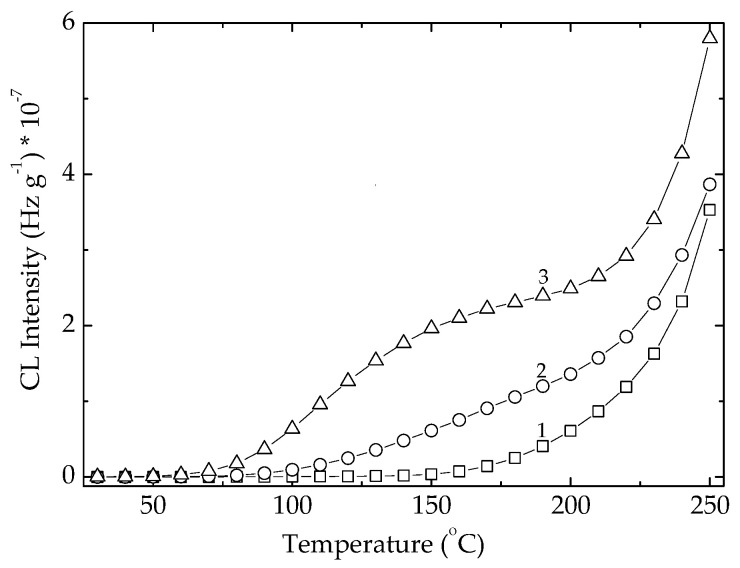
Non-isothermal CL spectra recorded on Ov-POSS powder at three irradiated doses. Heating rate: 10 °C min^−1^. (1) 0 kGy; (2) 25 kGy; (3) 100 kGy.

The non-isothermal CL spectrum recorded on row POSS powder (Figure 4) indicates the practical start of oxidation at around 140 °C. The continuous increase in the CL emission intensity defines a steady generation of radicals. The exposure of POSS powder to an intensive transfer of energy during the exposure to γ-radiation turns the oxidation start point onto lower temperatures (Figure 4).

At the irradiation dose of 25 kGy, the maximal dose for sterilization, the oxidation of powder becomes effective at 80 °C, while a higher dose of 100 kGy causes a strike reduction of the debut of oxidation down to 50 °C. At the same time, the corresponding increase of temperatures causing an advanced fragmentation rate is achieved. However, the ESR investigations did not reveal any signal so that we may presume that radical feature of the degradation intermediates of POSS is screened by the vinyl units in an electronic interaction with π bonds. An exponential dependence of the CL intensities on a certain moment of degradation (at 150 °C; Figure S1) supports this assumption and correlates with the increase of radical number on the radiation dose. The ascendant shape of this growth (Figure S1) is suggested by an electronic effect of vinyl groups on the corner carbon atoms. The deterioration of the basic POSS structure must be considered as the increasing measured CL intensities by the detection of oxygenated degradation products coming after the edge scission. However, the higher loading with POSS component grants an increase in the thermal stability of EPDM (Figure S2) that determines our continuing these investigations with a POSS concentration of 5% as an optimal filler content.

**Figure 5:**
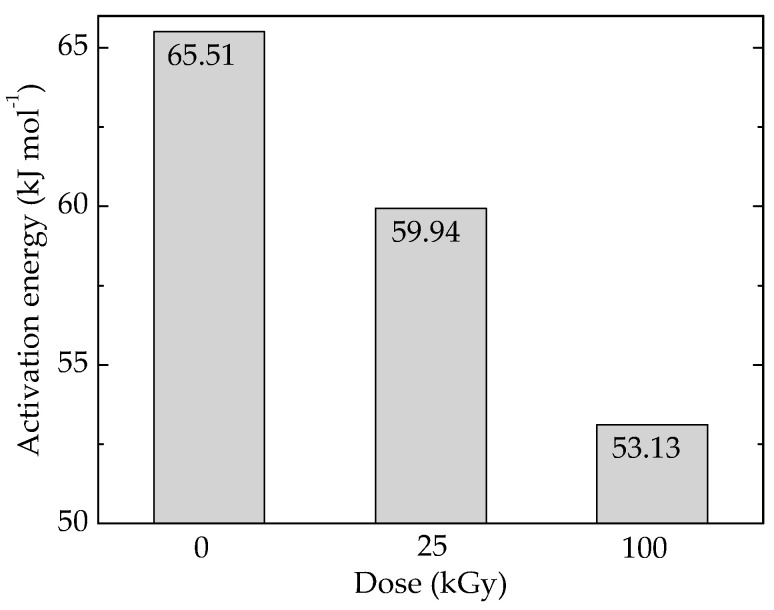
Activation energies values for the oxidation of pristine Ov-POSS.

The availability of POSS to the accelerated degradation is well depicted by the variation of activation energy required for this filler aging (Figure 5 and Table S1). The decrease in the values of activation energy is the result of breaking Si–O–Si that occurs on the edges of unity structural cell.

#### 3.1.2. Algal Extracts

**Figure 6:**
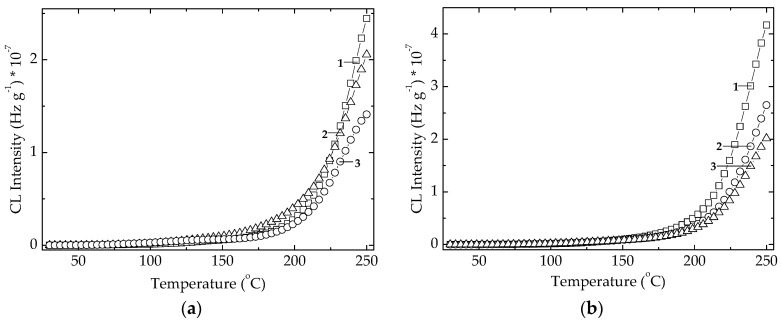
Non-isothermal CL spectra recorded on microalgal powders at three irradiated doses. (**a**) *Chlorella vulgaris*; (**b**) *Spirulina* *platensis*. (1) 0 kGy; (2) 25 kGy; (3) 100 kGy. Heating rate: 10 °C min^−1^.

The radiation effect on the studied algal extracts is characterized by the decrease in the CL intensities as the dose grows (Figure 6). The explanation of this abnormal improvement, that contradicts the destructive effect of high energy radiation, can be found in the conversion of active antioxidant components into other more efficient derivative compounds during the oxidation cascade advances [44]. The extract powders show small differences between their curves at various doses, but these discrepancies describe the intimate modifications in the component contributions inside polymer matrix. Accordingly, the amelioration of stabilization activities would be a meaningful effect that is a component of overall protection activity by overlapping effects.

### 3.2. Simultaneous Stability Effects of Additives in Pristine Polymer

**Figure 7:**
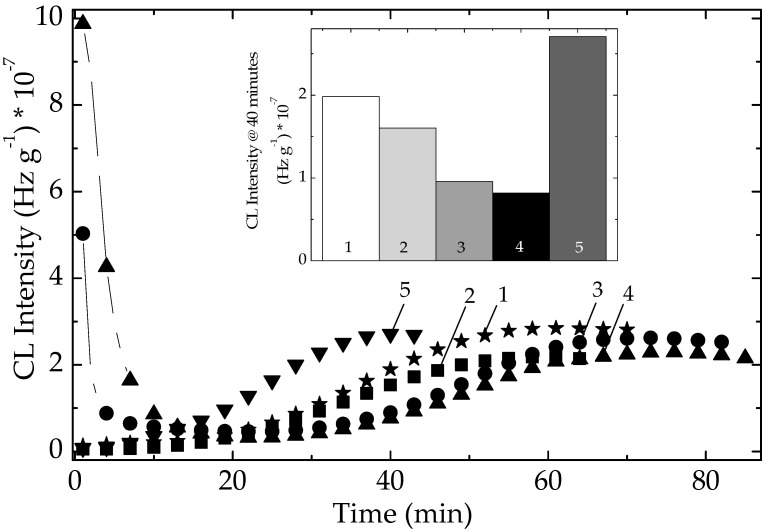
Isothermal CL spectra on unirradiated EPDM samples. Testing temperature: 170 °C. (1) Neat polymer; (2) EPDM/Ov-POSS 5; (3) EPDM/*Chlorella vulgaris*; (4) EPDM/*Spirulina* *platensis*; (5) EPDM/Ov-POSS 1.

**Figure 8:**
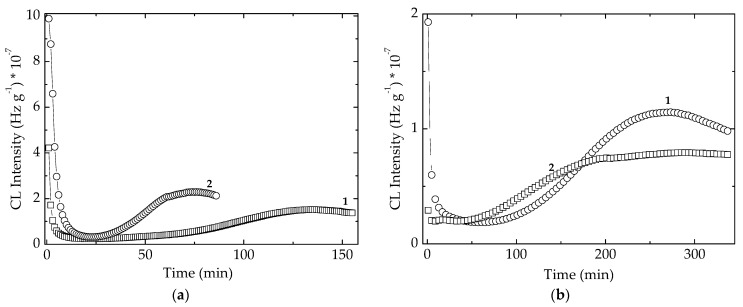
Comparative illustration of the isothermal CL spectra recorded on the unirradiated EPDM samples containing algal extracts and POSS. (**a**) *Spirulina platensis*; (**b**) *Chlorella vulgaris.* Dose: 0 kGy; Testing temperature: 170 °C. (1) POSS + algal extract; (2) algal extract.

The stability characteristics related to the sample compositions are correlated by the individual activities (Figure 7). The presence of some vegetal (for example, cellulose) traces in the algal extracts is illustrated by the high values of initial CL intensity, which are quickly consumed over the first 20 min of oxidation. The evolution of thermal degradation provides the suitable contributions of components for the protective activities. The inferior positions of CL emissions recorded on modified polymer (Figure 8) suggest the active participation of additives. The slower advance of oxidation in stabilized EPDM with algal extracts certifies the capability of these additives for an efficient protection of polymer material. The measure of protection capacities shown by various sample compositions is the value of time when the degradation reaches maximum intensity (Figure 8). The greater the maximum oxidation time, the more extended durability may be obtained. In spite of the generation of early formed hydroperoxides, the precursors of degradation products (Figure S3), the tested extracts show a continuous protection action. The evolutional comparison of increasing CL intensities on the high temperature range confirms the antioxidant potential of natural extract by which polyphenolic compounds prevent oxidation.

One appropriate feature for the applications of studied compositions in the manufacturing of plastics applications is the cooperation activities between POSS structure and the antioxidant components of algal extracts. Figure 8 recommends the concern paid for the extension of application range over several materials subjected to sustained aging.

### 3.3. Thermal Degradation of EPDM Composites

**Figure 9:**
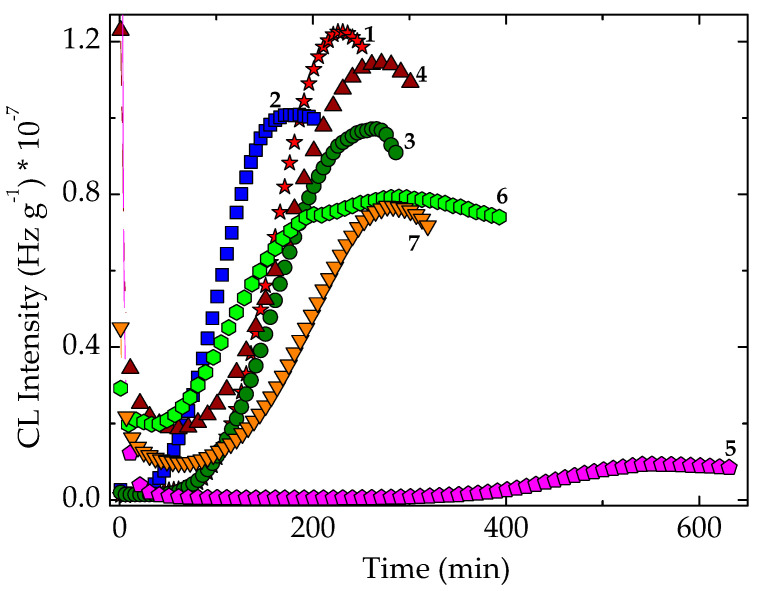
Isothermal spectra recorded at 150 °C on nonirradiated EPDM hybrids. (1) Neat polymer; (2) EPDM/Ov-POSS 1; (3) EPDM/Ov-POSS 5; (4) EPDM/*Chlorella vulgaris*; (5) EPDM/*Spirulina* *platensis*; (6) EPDM/Ov-POSS 5/*Chlorella vulgaris*; (7) EPDM/Ov-POSS 5/*Spirulina* *platensis*.

From Figure 9, the behavior analysis of stabilization efficiency of additives during the thermal oxidation of EPDM at 150 °C can be performed based on the different process evolution. The evident discordance is shown by the EPDM/*Spirulina* *platensis* that confirms the high efficiency of this extract for the screening oxidative degradation of host polymer. At this point, the presence of *SP* in the hybrid compositions exercises a stronger action in respect to the contribution of *CV*, which is also an appropriate factor in the attaining good stability parameters. The tested compositions, except EPDM/Ov-POSS 1 and, partially, EPDM/POSS/*Chlorella vulgaris*, present satisfactory kinetic parameters (oxidation induction time, maximum height reached at the end of oxidation and oxidation rate) that validate these hybrids as interesting formulations for the conservation of material integrity and oxidation states. The confirmation of this trend in the delaying of oxidation is addressed (Figure S4) for the active components of extracts which optimize the stability regime by their polyphenolic characteristics.

In accordance with the results from Figure 9, the increase in the degradation temperature creates a convenient situation, when the both types of additives cooperate. The illustration of this challenge is the activation energies that are necessary for the retardation of oxidation. These values (Table 1) are obtained by the isothermal CL determinations of thermal stability of EPDM assisted by algal extracts and Ov-POSS powder (Figure S5). The most suggestive values are shown for the material resistance at 50 °C (Table S1), the temperature possibly obtained during the prolonged exposure to the sun light. The detached group characterized by a raised durability consists of the hybrids with POSS 5 wt%, Spirulina, and their couple. This feature is obviously explained by the higher activation energies necessary for delaying degradation.

The oxidation strength of EPDM is intrinsically dependent on the structure and content of diene incorporated in the macromolecules. Consequently, the resistance against oxidation is characterized by various values of activation energies, whose values are increased when the polymer is protected by the oxidation inhibitors. Several spread values of activation energies for the oxidative degradation of EPDM have been reported, for example, 75 kJ mol^−1^ [45] and 162–226 kJ mol^−1^ [46]. Our figures are placed on the middle of this range, which is explained by the convenient content (5%) of diene and the medium content of propylene component (C2/C3 = 2/1). The mechanistic conformity of this performance is sustained by the influence of molecular structure, which defines the vulnerable spots, unsaturation from diene, and substituted carbon atoms from propylene moieties.

### 3.4. Radiation Degradation of Hybrids

The stabilization action of microalgal extracts has been previously demonstrated [29,47] on pristine EPDM substrates, in which evidence for their stability is associated with the local scavenging initial intermediates prior their interaction with diffused oxygen. The susceptibility on EPDM to the fast oxidation is counteracted by the additive involvement during propagation stage of aging. The exposure to γ-radiation initiates an accelerated oxidation due to the high local concentration of free radicals that are available to support the propagation of oxidation in the absence of any inhibitor. The inclusion of EPDM through the crosslinkable polymers characterized by the promotion of curing in opposition with degradation under radiation is evidenced by the contribution of additives to the growth of insoluble fraction (Table 2).

As the result of the protection of free radicals, the improvement of functional characteristics does not need the presence of crosslinker in the initial formulation as it would be required for the promotion of new crosslinks [48].

**Figure 10:**
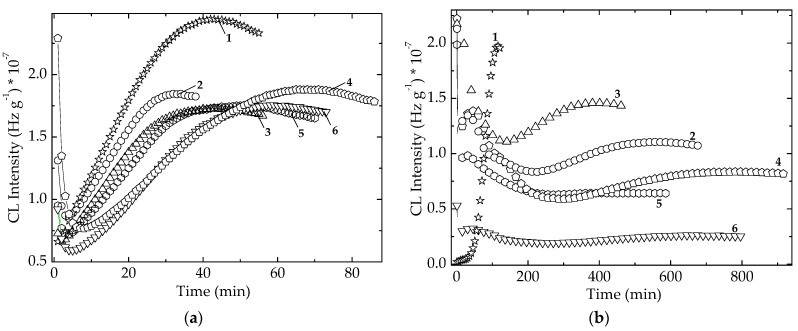
Isothermal spectra irradiated EPDM hybrids. (**a**) dose 25 kGy; temperature: 160 °C; (**b**) dose 100 kGy; temperature: 150 °C. (1) Neat polymer; (2) EPDM/Ov-POSS 5; (3) EPDM/*Chlorella vulgaris*; (4) EPDM/*Spirulina platensis*; (5) EPDM/Ov-POSS 5/*Chlorella vulgaris*; (6) EPDM/Ov-POSS 5/*Spirulina* *platensis*.

Figure 10 suggests the contribution of additives to the delay of degradation, which is specifically hindered in relation with the sample composition. It is obvious that all the CL curves in the both figures are underneath and on the right-hand side in respect to the position of the curve belonging to the neat material. The difference that exists between the two families of curves recorded at two different irradiation grades is their spread. If the low irradiation dose acts predominantly on the molecular chain scission accompanied by the inhibition of oxidation, and the CL isothermal curves are close to each other, at 100 kGy, or other superior doses, these curves are placed at various distances to one another. The crosslinking contribution of scission fragments is sustained by the stabilization efficiency that maintains the active free radicals far from their reactions with oxygen. The association of the two simultaneous processes, oxidation inhibition and crosslinking, determines the extension in the duration of aging and proffers an improvement of durability that is an important advantage for the activity effects. The exposure of EPDM simultaneously modified with Ov-POSS and algal extracts proves the co-operation by different stabilization mechanisms between the two additives that complete each other in the propitious decay of free radicals.

**Figure 11:**
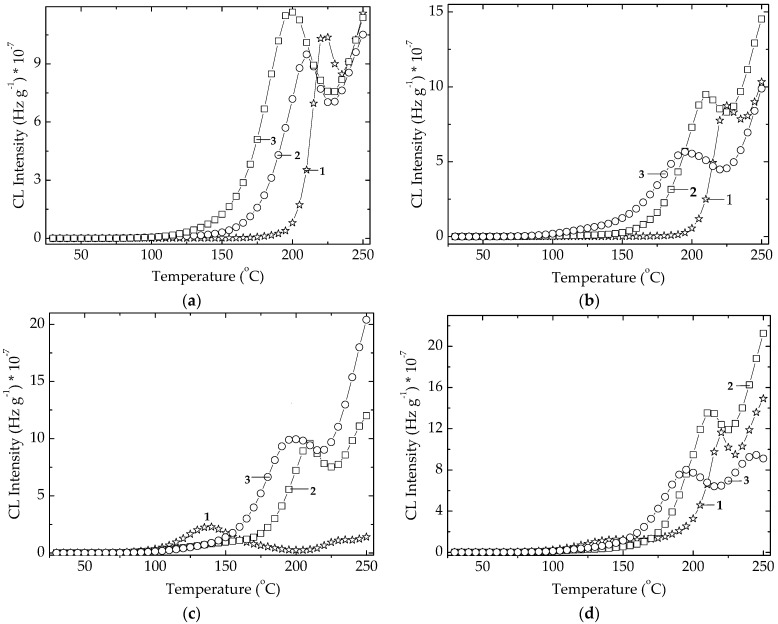
Isothermal spectra record on EPDM hybrids aged by γ-irradiation. (**a**) Control; (**b**) EPDM/Ov-POSS 5; (**c**) EPDM/*Spirulina* *platensis*; (**d**) EPDM/*Chlorella vulgaris.* Doses: (1) 0 kGy; (2) 25 kGy; (3) 100 kGy. Heating rate: 5 °C min^−1^.

The degradation conditions that influence the life of packaging products are characterized by the diffusion feature of materials and the hindering of oxidation due to the antioxidative effects. The evolution of oxidation state in degrading polymer phase is depicted by the temperature (onset oxidation temperature, OOT), when the oxidation of materials begins to be measurable (Figure 11). The neat EPDM presents the lowest OOT values because it is not protected by any compound. The presence of Ov-POSS (5 phr) induces a decrease in the oxidation level at the temperatures exceeding 150 °C, while the microalgal extracts show a shoulder at 140 °C in unirradiated specimens. The γ-exposure erases it by the structural damage of cellulose traces existing in microalgal powders. These two stabilizers act differently. If the unirradiated sample containing *Spirulina* *platensis* keeps oxidation degree at very low level (Figure 11c), and the oxidation on the higher values range starts earlier, the specimen containing *Chlorella vulgaris* begins to be deeply oxidized at about 150 °C, but the progress of degradation advances more slowly than in the analogous sample with *SP*. This aspect is good evidence for the stabilization of polymers with microalgal extracts, when they are subjected to γ-ray sterilization.

The comparison between the radiation resistances of modified EPDM by the increase in the oxidation strength of γ-irradiated polymer samples orders these formulations in the following order:

Ov-POSS 5 > Chlorella vulgaris > Spirulina platensis.

However, the discrepancy between the protection activities of the two studied microalgal extracts is minimal. Their applications as the agents for oxidation preventing are recommended, as well as the analogous contribution of Ov-POSS.

The effect of heating rate on the progress of oxidation in EPDM as an example of polyolefins may be analyzed by the heights of oxidation maximum in the temperature range from 140 °C to 160 °C. The more intense the heat transfer, the higher the CL emission intensity. At the same time, the greater heating rate shifts this maximum toward higher temperatures, which may be explained by the impossibility of degradation fragments to meet suddenly with diffuse oxygen.

The non-isothermal spectra recorded during the thermal oxidation of EPDM modified with microalgal extracts also reveals their relevant contributions to the prevention of oxidation at the temperatures exceeding 150 °C (Figure 11 and Figure S6). While the unprotected material begins to be aged at lower temperatures, the modified EPDM becomes vulnerable at higher temperature, and the durability is significantly improved.

### 3.5. Evaluation of Durability

The resistance of EPDM to the thermal oxidation is dependent on the restraining efficiency of additive. Table 3 illustrates the contribution of filler to the delay of aging. While the values of durability are significantly higher when Ov-POSS, *Chlorella vulgaris*, and *Spirulina* *platensis* act upon oxidation, the slopes of lines describing the evolution of durability are greater than the similar value obtained for pristine polymer.

### 3.6. Evaluation of Crosslinking

The competition between the scission and crosslinking of polymer macromolecules is the factor that determines the occupied place in the two separate groups: degradable or crosslinkable compounds, respectively. The evolution of insoluble content of polymers allows characterizing the stability under γ-irradiation. From Table 2 the last column describes the contribution of additives to the promotion of radical recombination due to the ratio p/q, the ratio between the radiochemical yields of scission, and crosslinking, respectively. The decrease of these values indicates the higher proportion of crosslinking events, which is closely related to the stability efficiencies of oxidation protectors. The highest figures of gel fractions in the EPDM samples containing Ov-POSS/microalgal extracts irradiated at 100 kGy prove the synergism of these antioxidant couples. The antirad action of the two stabilizing couples depicts the co-operation of components and the stimulation of high radiation strength by blocking the progress in the structural failure.

The improvement in the material integrity after the action of degradation factors (heat and ionizing radiation) is achieved not only by the inactivation of free radical reactivity but also by the increase in the insoluble fraction, which is responsible by the reconstruction of macromolecules and prevents the diffusion of molecular oxygen that feeds degradation.

## 4. Discussion

The degradation of EPDM is a prominent process, where an appropriate compound for the delaying oxidation is absent. The addition of a certain inorganic filler (Ov-POSS) and microalgal extracts proves their prevention potential in respect to oxidative degradation. If the protection mechanisms of Ov-POSS and microalgal extracts are totally different (Figure 1), their contributions are notably evidenced by several reliable investigations. The chemiluminescence determinations reveal the proper effect of protection on the kinetic parameters (oxidation induction time in isothermal CL spectra, onset oxidation temperature in non-isothermal CL determinations, activation energy). The inhibition of oxidation shown by the studied additives leads to a high-performances that are related by the material durability. This feature is attained due to the efficient restriction of oxidation and to the increase in the gel content by γ-irradiation. It means that the added compounds in the formulation of EPDM formulations bring about a useful improvement in the degradation resistance by the stimulation of the recombination of free radicals prior to their oxidation and their inactivation by scavenging inside the spatial configuration (Ov-POSS) or by proton replacing (microalgal extracts). The host polymer material undergoes an appropriate amelioration of withstanding capacity against oxidative aging, when the two categories of compounds provide antioxidant couples.

The stabilization efficiency places the investigated compounds in a certain order, which explains their usefulness for the preservation of product entirety. The highest stability is achieved by means of Ov-POSS (5 phr). A good durability performance is also obtained by the presence of microalgal extracts (2 phr), but this modification has an enormous advantage consisting of their encouraging feature of biocompatibility. The proposed procedure for the manufacture of higher durability of EPDM-based products involves the advantageous effects related to the enlarging the application areas due to the satisfactory consequences on the delaying oxidation, on the maintaining product integrity, on the low costs of item production.

The co-operation actions of the two kinds of oxidation protectors are based on the complementary effects that involve two different manners for the inactivation of free radical reactivity in respect to molecular oxygen. If a γ-irradiation processing is applied, the material stability increases accordingly.

Microalgae extracts are a potential source for several activities, including the improvement of the applications involving bioactive compounds [49,50]. The antioxidant properties as they were illustrated in this paper may be obtained [51], characterized [52,53], and applied [54] as the result of multicomponent content and easy availability.

## 5. Conclusions

This paper presents the satisfactory improvement of thermal and radiochemical stabilities by the addition of some appropriate compounds, vinyl-POSS and microalgae: *Chlorella vulgaris* and *Spirulina platensis*. They act efficiently on the delay of oxidative degradation by the blocking the reactivity of free radicals. The chemiluminescence determinations, as well as the crosslinking investigation, prove the involvement of these structures in the withdrawing degradation intermediates from their oxidation. While the isothermal CL measurements allow remarking on the increase in the values of oxidation induction time for all modified formulation, except the composition of EPDM samples containing Ov-POSS 1 phr, the non-isothermal CL measurements define the contribution of studied additives to the delaying oxidation by the higher figures of onset oxidation temperatures. These kinds of assays make differences between the two microalgal extracts that show a shoulder at about 100–120 °C and the retarded evolution of oxidation on the higher temperature range. The conclusive proof of protection features is the values set of activation energies required by the degradation of EPDM component, and the increase in the insoluble fraction of γ-irradiated EPDM-based samples undoubtedly sustain the decay of free radicals by recombination, instead of their oxidation. The association of one tested microalgal extract with vinyl-POSS is a profitable solution for the achieving the high-performance stability with which the products may have an extended durability.

The degree of oxidative degradation in EPDM-based materials is effectively diminished by the two different withdrawing matters: the blocking of radical intermediates inside the free hole of tetrahedral configuration (Ov-POSS) or the scavenging free radicals by the alkoxyl structure of phenolic components of microalgal extracts. The coupling of these stabilization mechanisms offers a proper version of stability improvement for polymer materials.

The γ-irradiation assay demonstrates that the proposed solutions for the manufacture of high-performance materials by the presence of Ov-POSS and/or microalgal extracts may be acceptable for the products subjected to γ-processed or γ-sterilized articles.

## Figures and Tables

**Table 1:** Activation energies of thermal oxidation of unirradiated EPDM-based hybrids.

Additive	Oxidation Induction Time (min)			Correlation Factor	Activation Energy (kJ mol^−1^)
	160 °C	170 °C	180 °C		
none	110	45	25	0.99442	120.8
POSS 1	50	20	10	0.99723	102.0
POSS 5	89	30	15	0.99596	150.7
*Chlorella vulgaris*	130	62	28	0.99944	125.1
*Spirulina* *platensis*	300	130	55	0.99990	139.5
POSS 5/*Chlorella vulgaris*	90	40	20	0.99948	122.6
POSS 5/*Spirulina* *platensis*	183	62	31	0.99364	145.0

**Table 2:** Gel fractions in irradiated EPDM-based hybrids.

Sample	Gel Fraction			Correlation Factor	p/q
	D 25	D 50	D 100		
Neat	3.5	11	23	0.99587	1.50
POSS 5	5.1	16	28	0.97873	1.48
*Chlorella vulgaris*	5.5	20	31	0.99675	1.39
*Chlorella vulgaris*	5.4	22	35	0.99206	1.33
POSS 5/*Chlorella vulgaris*	6.4	30	46	0.99911	1.12
POSS 5/*Spirulina* *platensis*	7.2	45	53	0.98087	0.86

**Table 3:** Durability of EPDM to the thermal degradational at low temperatures.

Additive	Durability (h)		
	25 °C	30 °C	50 °C
none	*Y = −28.5 + 14.55 X*		
	2939.2	1303.3	63.3
POSS 1	*Y = −24.7 + 12.27 X*		
	191.7	97.8	10.1
Ov-POSS 5	*Y = −35.9 + 17.47 X*		
	1,179,693.0	44,240.0	1090.8
*Chlorella vulgaris*	*Y = −29.8 + 15.05 X*		
	4081.5	1514.7	87.6
Ov-POSS 5/*Chlorellavulgaris*	*Y = −29.6 + 14.75 X*		
	2940.8	1294.8	64.6
*Spirulina* *platensis*	*Y = −33.0 + 16.78 X*		
	26,629.1	10,833.9	342.6
Ov-POSS 5/*Spirulina* *platensis*	*Y = −35.1 + 17.44 X*		
	55,964.5	21,746.5	607.5

## Data Availability

Not applicable.

## Supplementary Materials

The following are available online at https://www.mdpi.com/article/10.3390/polym14010187/s1, Figure S1: Evolution of CL emission for Ov-POSS powder at 150 °C measured during nonisothermal determination, heating rate: 10 °C min^−1^, Figure S2: Isothermal CL spectra recorded on two different EPDM hybrids, (1) EPDM/Ov-POSS 1; (2) EPDM/Ov-POSS 5, temperature 170 °C; dose 0 kGy, Figure S3: Nonisothermal CL records on different EPDM hybrids, (1) EPDM neat; (2) EPDM/Ov-POSS 5; (3) EPDM/*Spirulina* *platensis*;(4) EPDM/*Chlorella vulgaris*; (5) EPDM/Ov-POSS 1, dose: 0 kGy, heating rate: 15 °C min^−1^, Figure S4: Nonisothermal CL records on different EPDM hybrids, (1) EPDM neat; (2) EPDM/Ov-POSS 5; (3) EPDM/*Spirulina* *platensis*;(4) EPDM/*Chlorella vulgaris*; (5) EPDM/Ov-POSS 1, dose: 0 kGy, heating rate: 5 °C min^−1^, Figure S5. Isothermal CL spectra recorded on unirradiated EPDM hybrid composites: (1) 160 °C, (2) 170 °C, (3) 180 °C, (a) pristine EPDM; (b) EPDM/POSS 1; (c) EPDM/*Chlorella vulgaris*; (d) EPDM/*Spirulina* *platensis*; (e) EPDM/POSS 5/*Chlorella vulgaris*; (f) EPDM/POSS/*Spirulina* *platensis;* (g) EPDM/POSS 5, Figure S6: Nonisothermal CL spectra ascribed to EPDM modified with algal extract, (1) 0 kGy; (2) 25 kGy; (3) 100 kGy, EPDM/POSS 5/*Chlorella vulgaris*: (a) 3.7 °C min^−1^; (b) 5.0 °C min^−1^; (c) 10.0 °C min^−1^; (d) 15.0 °C min^−1^, EPDM/POSS 5/ *Spirulina platensis*: (e) 3.7 °C min^−1^; (f) 5.0 °C min^−1^; (g) 10.0 °C min^−1^; (h) 15.0 °C min^−1^, Table S1. OOT values and their correlation.
