# Supplementary material for: Stability Efficiencies of POSS and Microalgae Extracts on the Durability of Ethylene-Propylene-Diene Monomer Based Hybrids
Source: Polymers (Basel). 2022 Jan 3;14(1):187. doi: 10.3390/polym14010187 (PMC8747662; doi:10.3390/polym14010187)
Supplement: Supplementary file 1 [file polymers-14-00187-s001.zip › polymers-1459475-supplementary.pdf]

Figures

Figue S1. Evolution of CL emission for Ov-POSS powder at 150 ºC measured during

nonisothermal determinations.

Heating rate: 10 ºC min^{-1}.

Figure S2. Isothermal CL spectra recorded on two different EPDM hybrids

(1) EPDM/Ov-POSS 1; (2) EPDM/Ov-POSS 5

Temperature 170 ºC; dose 0 kGy.

Figure S3. Nonisothermal CL records on different EPDM hybrids

(1) EPDM neat; (2) EPDM/Ov-POSS 5; (3) EPDM/*Spirulina platensis*;

(4) EPDM/*Chlorella vulgaris*; (5) EPDM/Ov-POSS 1.

Dose: 0 kGy. Heating rate: 15 ºC min^{-1}

Figure S4. Nonisothermal CL records on different EPDM hybrids

(1) EPDM neat; (2) EPDM/Ov-POSS 5; (3) EPDM/*Spirulina platensis*;

(4) EPDM/*Chlorella vulgaris*; (5) EPDM/Ov-POSS 1.

Dose: 0 kGy. Heating rate: 5 ºC min^{-1}

Figure S5. Isothermal CL spectra recorded on unirradiated EPDM hybrid composites

(1) 160 ºC, (2) 170 ºC, (3) 180 ºC.

(a) pristine EPDM; (b) EPDM/POSS 1; (c) EPDM/*Chlorella vulgaris*;

(d) EPDM/*Spirulina platensis*; (e) EPDM/POSS 5/*Chlorella vulgaris*;

(f) EPDM/POSS/*Spirulina platensis;* (g) EPDM/POSS 5.

Figure S6. Nonisothermal CL spectra ascribed to EPDM modified with algal extract

(1) 0 kGy; (2) 25 kGy; (3) 100 kGy

EPDM/POSS 5/*Chlorella vulgaris*: (a) 3.7 ºC min^{-1}; (b) 5.0 ºC min^{-1};

(c) 10.0 ºC min^{-1}; (d) 15.0 ºC min^{-1}.

EPDM/POSS 5/ *Spirulina platensis*: (e) 3.7 ºC min^{-1}; (f) 5.0 ºC min^{-1};

(g) 10.0 ºC min^{-1}; (h) 15.0 ºC min^{-1}.

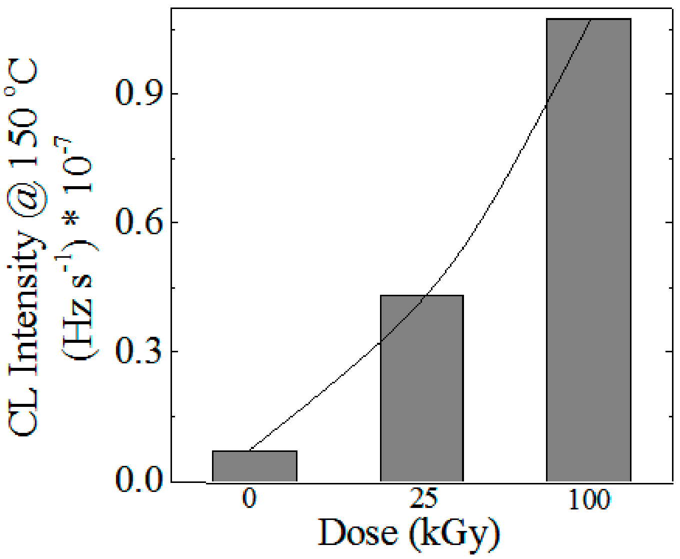

Figure S1.

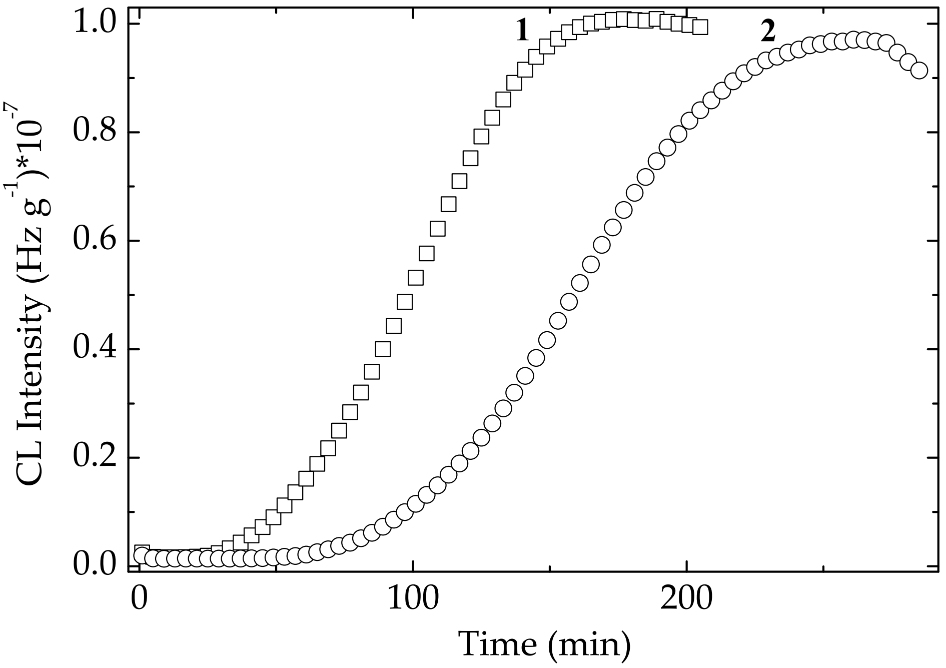

Figure S2

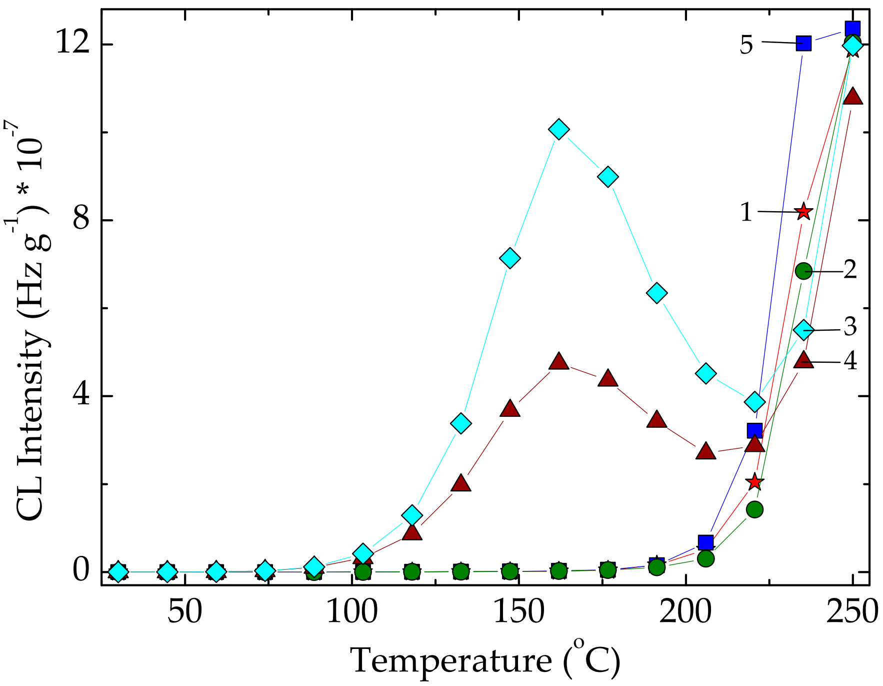

Figure S3.

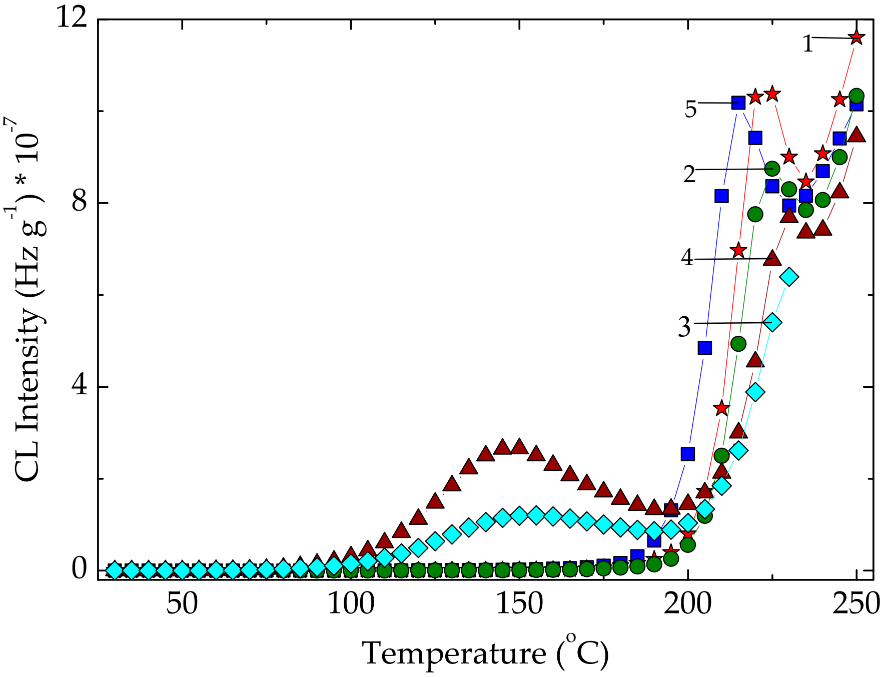

Figure S4.

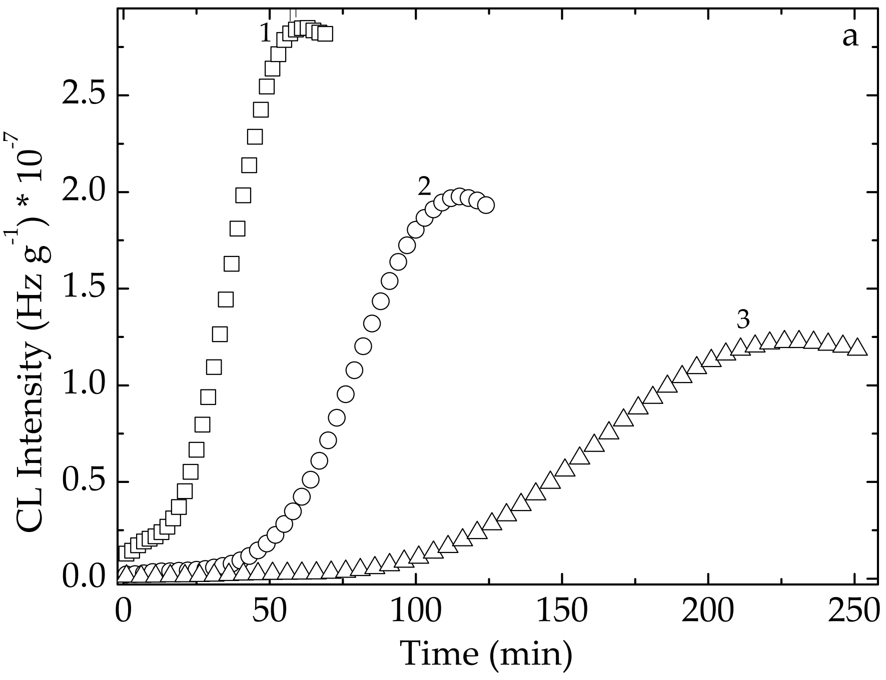
CL Intensity (Hz g-1) * 10-7
Time (min)
a
1
2
3
0.0
0.5
1.0
1.5
2.0
2.5
0
50
100
150
200
250

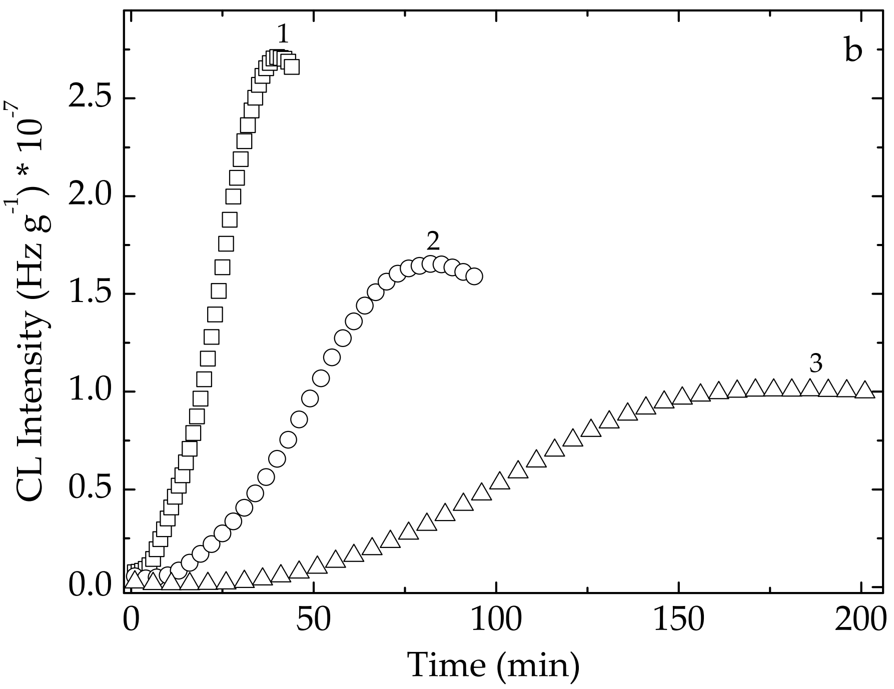
CL Intensity (Hz g-1) * 10-7
Time (min)
b
1
2
3
0.0
0.5
1.0
1.5
2.0
2.5
0
50
100
150
200

Figure S5.

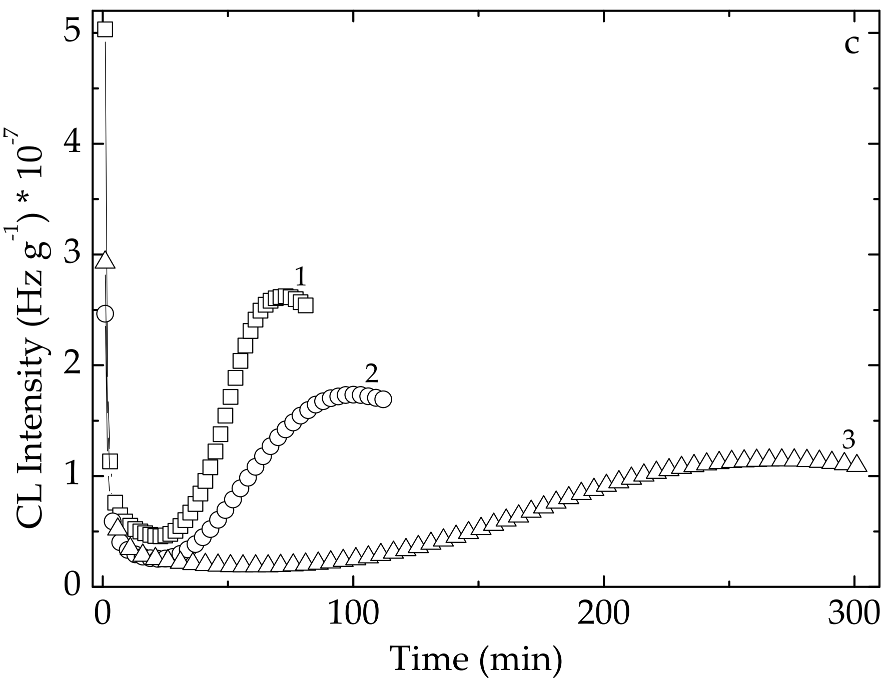

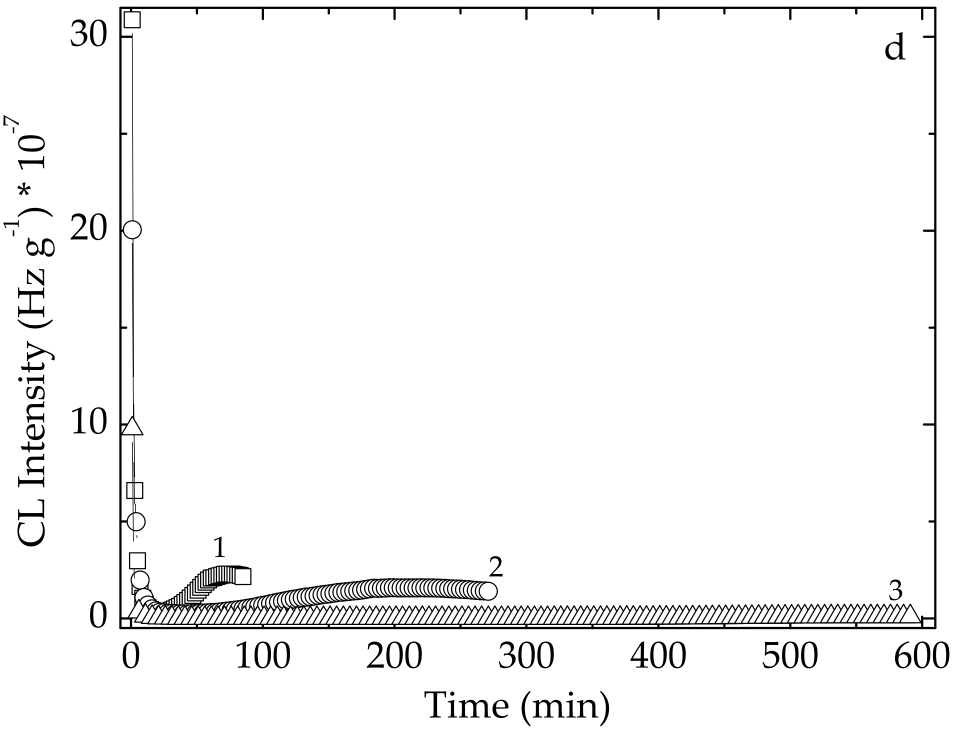

Figure S5. To be continued

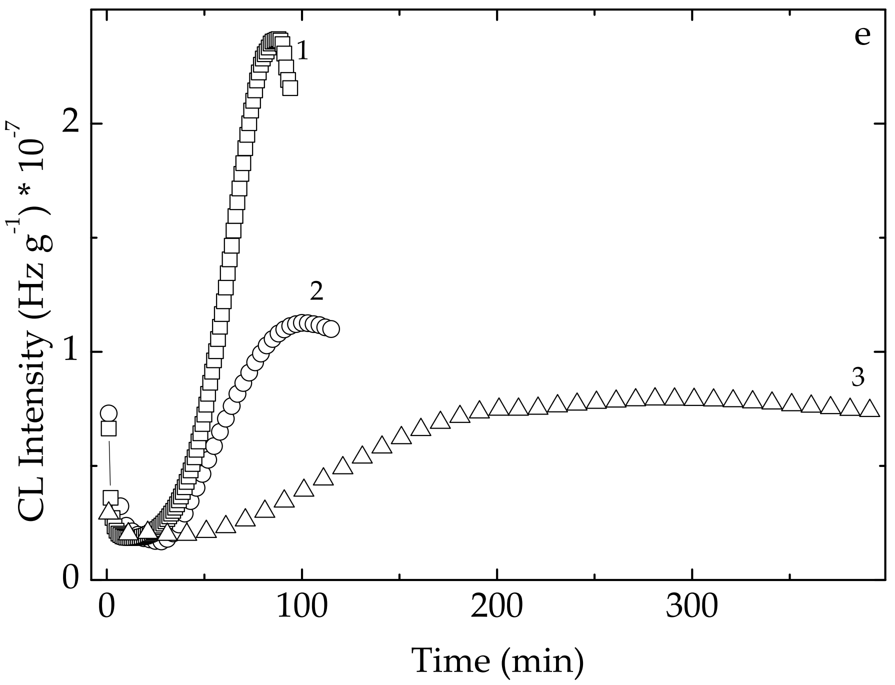

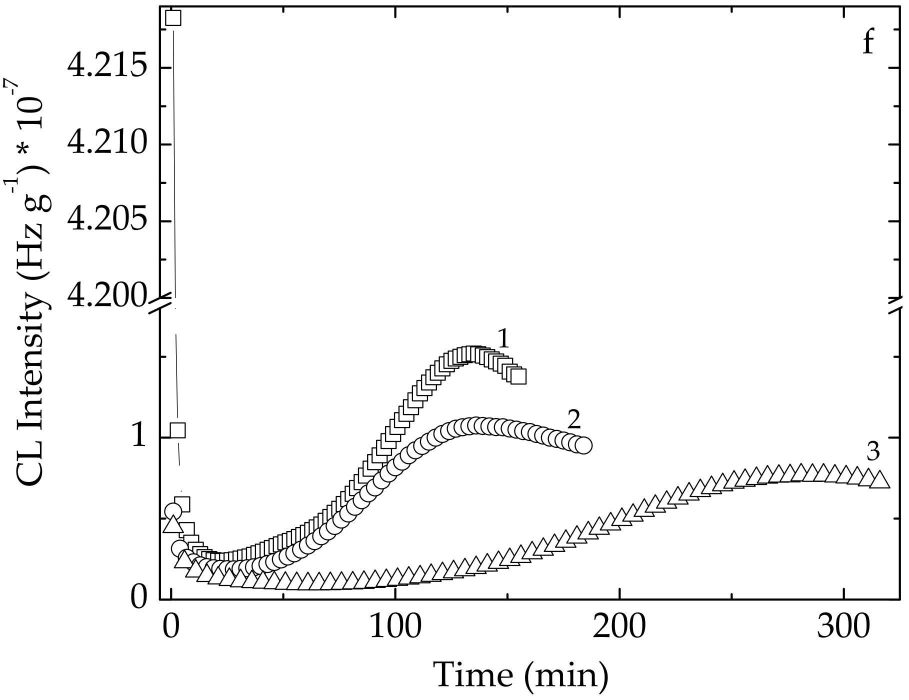

Figure S5. To be continued

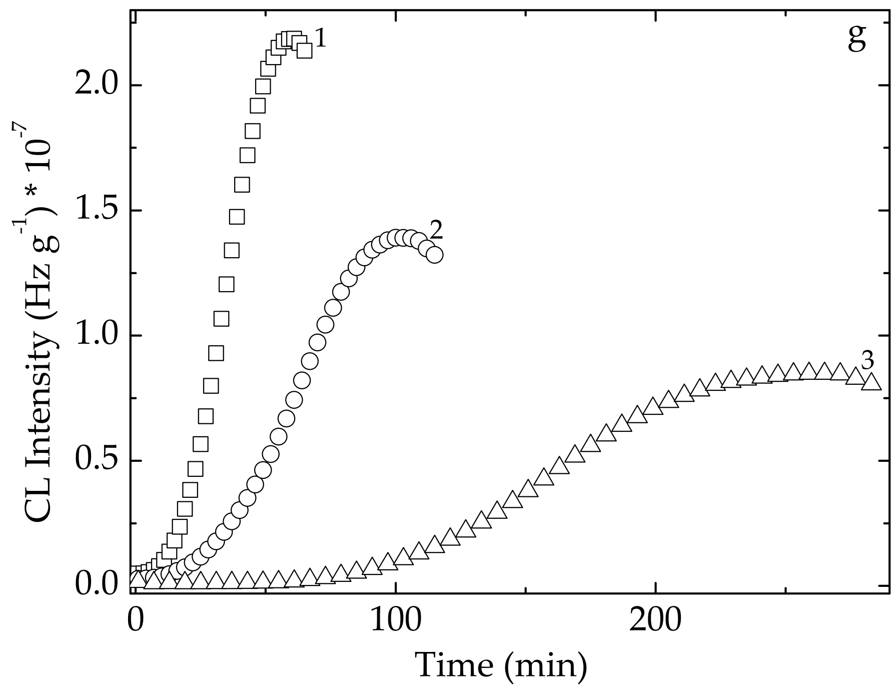

Figure S5. To be continued

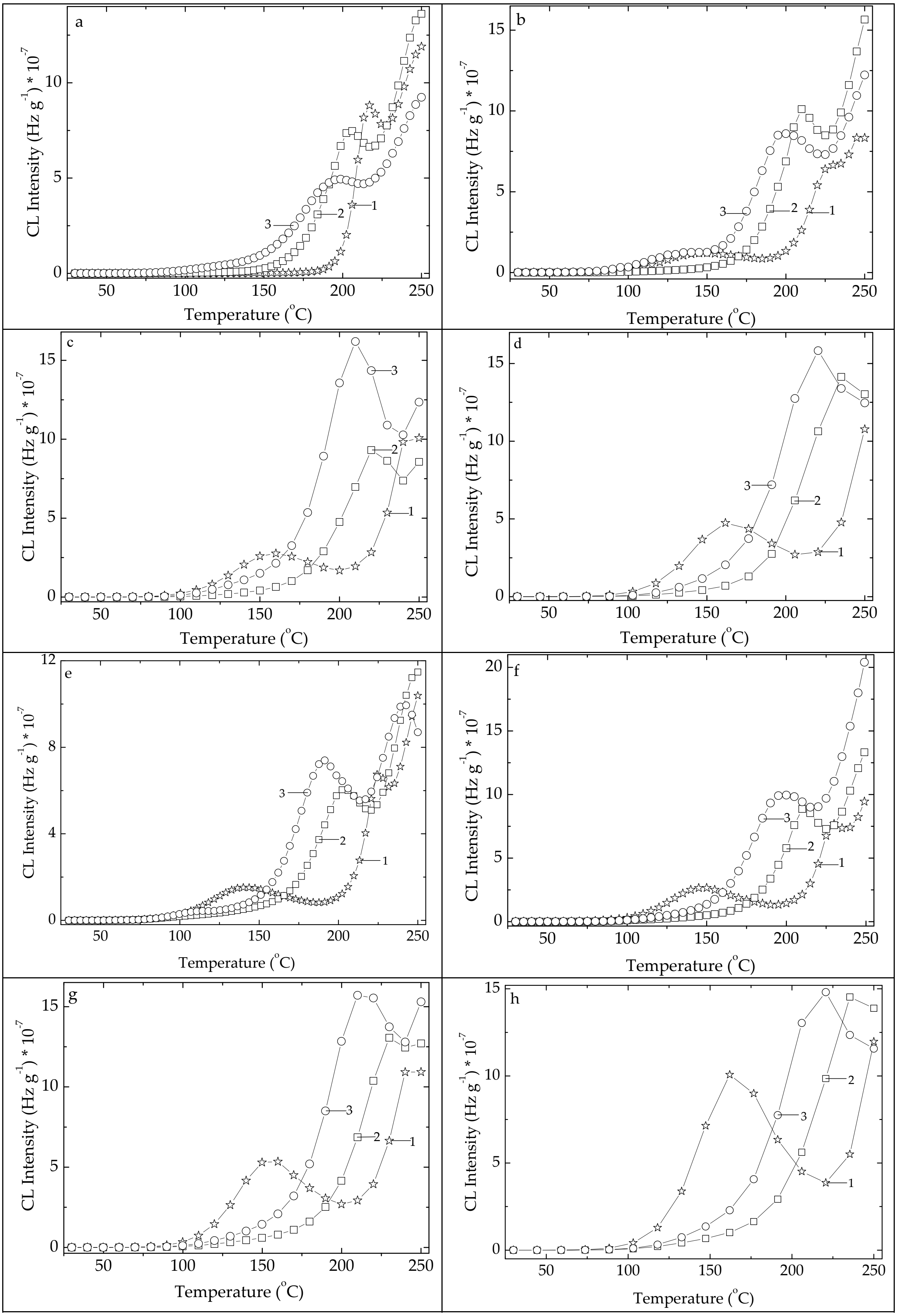

Figure S6.

Table

Table S1. OOT values and their correlation

Dose (kGy)	OOT (K)				Correlation factor
	3.7 ^{o}C min^{-1}	5.0 ^{o}C min^{-1}	10.0 ^{o}C min^{-1}	15.0 ^{o}C min^{-1}	
0	423	433	448	453	0.98287
25	418	428	443	451	0.99240
100	413	423	438	450	0.99578
